# Prediction of key genes and pathways involved in trastuzumab-resistant gastric cancer

**DOI:** 10.1186/s12957-018-1475-6

**Published:** 2018-08-22

**Authors:** Chaoran Yu, Pei Xue, Luyang Zhang, Ruijun Pan, Zhenhao Cai, Zirui He, Jing Sun, Minhua Zheng

**Affiliations:** 1 Department of General Surgery, Ruijin Hospital, Shanghai Jiao Tong University, School of Medicine, Shanghai, 200025 People’s Republic of China; 2 Shanghai Minimally Invasive Surgery Center, Ruijin Hospital, Shanghai Jiao Tong University, School of Medicine, Shanghai, 200025 People’s Republic of China

**Keywords:** Differentially expressed genes, Gene ontology, KEGG pathway, Gastric cancer, Trastuzumab, Resistance, Protein-protein interaction

## Abstract

**Background:**

Trastuzumab has been prevailingly accepted as a beneficial treatment for gastric cancer (GC) by targeting human epidermal growth factor receptor 2 (HER2)-positive. However, the therapeutic resistance of trastuzumab remains a major obstacle, restricting the therapeutic efficacy. Therefore, identifying potential key genes and pathways is crucial to maximize the overall clinical benefits.

**Methods:**

The gene expression profile GSE77346 was retrieved to identify the differentially expressed genes (DEGs) associated with the trastuzumab resistance in GC. Next, the DEGs were annotated by the gene ontology (GO) and Kyoto Encyclopedia of Genes and Genomes (KEGG) pathways. The DEGs-coded protein-protein interaction (PPI) networks and the prognostic values of the 20 hub genes were determined. Correlation of the hub genes were analyzed in The Cancer Genome Atlas. The prognostic values of hub genes were further validated by Kaplan-Meier (KM) plotter.

**Results:**

A total of 849 DEGs were identified, with 374 in upregulation and 475 in downregulation. Epithelium development was the most significantly enriched term in biological processes while membrane-bounded vesicle was in cellular compartments and cell adhesion molecular binding was in molecular functions. Pathways in cancer and ECM-receptor interaction were the most significantly enriched for all DEGs. Among the PPI networks, 20 hub genes were defined, including CD44 molecule (CD44), HER-2, and cadherin 1 (CDH1). Six hub genes were associated with favorable OS while eight were associated with poor OS. Mechanistically, 2′-5′-oligoadenylate synthetase 1, 3 (OAS1, OAS3) and CDH1 featured high degrees and strong correlations with other hub genes.

**Conclusions:**

This bioinformatics analysis identified key genes and pathways for potential targets and survival predictors for trastuzumab treatment in GC.

**Electronic supplementary material:**

The online version of this article (10.1186/s12957-018-1475-6) contains supplementary material, which is available to authorized users.

## Background

Gastric cancer (GC) remains one of the leading common causes for cancer-related mortality and major global heath challenges [1–4]. Despite the incidence declining in industrialized nations, most new cases are occurred in South America, East Asia, and Eastern Europe [2, 5]. Surgery is the primary treatment for resectable GC [6]. However, the dissection extent of lymph node (D1, D2) remains controversial [3]. Kang et al. reported 46.5% patients who underwent curative surgery experienced recurrence, and half of the recurrence occurred in less than 3 years [7]. In the Dutch Gastric Cancer Group (DGCG) trial, 65% curative resected patients experienced recurrence with 30% overall survival (OS) for D1 and 35% for D2 [8]. Consistently, the Medical Research Council (MRC) trial reported a 34% 5-year OS [9]. Noteworthy, the inclusion of targeted drugs, such as angiogenesis inhibitors (ramucirumab) and epidermal growth factor receptor (EGFR) antibodies (nimotuzumab), have shown encouraging therapeutic benefits in GC patients [10, 11].

Trastuzumab, a monoclonal antibody targeting epidermal growth factor receptor 2 (HER2) in breast cancer [12], was also among the promising therapeutic management to the GC patients with HER2-positive [13, 14]. It eliminated the activity of HER2 receptor and weakened subsequent multiple signaling pathways [15]. The first randomized prospect trial had shown that a triplet regimen of trastuzumab, cisplatin, and a fluoropyrimidine significantly improved the median OS of GC with HER2 overexpression or amplification [13]. In fact, secondary resistance was acquired within a median of two therapeutic cycles [16]. Until now, the resistance to trastuzumab in GC remains a major obstacle with limited clinical benefits. Efficient biomarkers and underlying mechanism are yet to be fully elucidated.

Hereby, potential biomarkers and pathways associated with trastuzumab resistance were investigated in GC cell lines by the gene expression profile, GSE77346 [17], from the Genetic Expression Omnibus (GEO) database (http://www.ncbi.nlm.nih.gov/geo/). The prognostic values of the biomarkers and potential mechanisms were assessed.

## Methods

### Gene expression profile from GEO database

The gene expression profile, GSE77346, was retrieved from the Gene Expression Omnibus (GEO) database (http://www.ncbi.nlm.nih.gov/geo/) [18]. The profile was generated by GPL10558, Illumina Human 48 K gene chips (Illumina HumanHT-12 V4.0 Expression BeadChip). The GSE77346 dataset consisted of one trastuzumab-sensitive NCI-N87 cell line and four trastuzumab-resistant cell lines (N87-TR1, N87-TR2, N87-TR3, N87-TR4). Briefly, all the cell lines were maintained in Roswell Park Memorial Institute (RPMI) 1640 medium with 10% heat-inactivated FBS. The green fluorescent protein (GFP) +/luciferase+ NCI-N87 cell lines were harvested and injected into the gastric walls of a nude mice. The tumor-bearing mice were received 20 mg/kg trastuzumab i.p. twice per week when the resulting tumors were detectable (Living Image Software program, Xenogen). The trastuzumab treatments were stopped when the tumors were relapsed. By repeated GFP flow cytometric sorting (FACSAria II sorter, Becton Dickinson), four trastuzumab-resistant cell lines were established [17]. Next, total RNA was retrieved by TRIzol reagent (Ambion, Warrington, UK). The synthesis of biotinylated cRNA (Illumina TotalPrep RNA Amplification Kit, Ambion) and the hybridization (Human HT-12 V4 BeadChip) were performed according to the manufacturer protocols. Probe intensity was obtained and normalized by the Illumina GenomeStudio software (Genome Studio V2011.1) [17]. The gene expression profiles GSE13861, including 84 samples (65 tumors and 19 normal tissues), were used for investigation of mRNAs expression of the hub genes between tumor and normal tissues (Illumina Human V3) [19]. For external validation on gene expression profiles with other target drugs, we further included GSE19043 and GSE95414. GSE19043 contained 21 samples from DiFi and GTL-16 cell lines, of which biological triplicates of DiFi cells with gefitinib (EGFR inhibition) and DMSO (control) were used in this study for validation. The platform was GPL5104, Sentrix HumanRef-8 v2 Expression BeadChip [20]. GSE95414 contained one parental NCI-N87 cell line and one trastuzumab-DM1 (T-DM1, trastuzumab emtansine)-resistant cell line. T-DM1 is designed to achieve a combinational therapy of trastuzumab and DM1 (a potent microtubule-disrupting drug, a maytansine derivative) [21]. The RNA was processed by Human Transcriptome Array 2.0 arrays (Affymetrix, GPL17586). Given the absence of biological replicates, the fold change between the T-DM1-resistant cell line and parental cell line was used for investigation (original study of GSE95414 is not yet published).

### Data processing on DEGs

The differentially expressed genes (DEGs) between the trastuzumab-resistant cell lines and sensitive control were identified by the GEO2R analytical tool [22]. Benjamini and Hochberg method was used for false discovery rate (FDR). The cut-off values of DEGs were defined as adj.*p* value < 0.05 and log2 fold change (log FC) > 2 or < − 2. The DEG expression data were processed for a bidirectional hierarchical clustering plot (FunRich, http://www.funrich.org) [23].

### Gene ontology and pathway analysis of DEGs

The Database for Annotation, Visualization, and Integrated Discovery (DAVID, http://david.abcc.ncifcrf.gov/) was employed for the gene ontology (GO) consortium reference, including biological processes (BP), cellular components (CC), and molecular functions (MF) [24, 25]. In addition, DAVID was also employed for pathway enrichment annotations with the data resources from Kyoto Encyclopedia of Genes and Genomes (KEGG, http://www.genome.jp/kegg/) pathway enrichment analysis [24, 26].

### Protein-protein interaction (PPI) networks and module analysis

The interaction networks of the DEG-coded proteins were determined by the Search Tool for the Retrieval of Interacting Genes/Proteins (STRING, http://www.string-db.org/) [27]. Node degree ≥ 5 was defined as the cut-off values for further PPI networks visualization by Cytoscape software (version 3.6.0; http://www.cytoscape.org/) [28]. The Molecular Complex Detection (MCODE) program embedded in Cytoscape was used to subcluster the PPI networks with predefined cutoff criterions (max. depth = 100, node score = 0.2 and *k*-score = 2) [29]. Hub genes were defined by the degree value (paired connections between each node). In addition, the betweenness centrality (defining the fraction of shortest paths involved in a given node) of the hub genes were also added.

### Survival analysis of the hub genes

Kaplan-Meier (KM) plotter enables comprehensive analysis of the prognostic values among lists of genes in various cancers based on multiple genomic profiles, including GSE14210, GSE15459, GSE22377, GSE29272, GSE51105, and GSE62254 [30]. The prognostic values of overall survivals (OS) for hub genes were displayed with the hazard ratios (HR) and log-rank *p* values.

### Hub genes correlation in TCGA

The gene expression profiling interactive analysis (GEPIA, http://gepia.cancer-pku.cn) was established for customized genomic analysis based on The Cancer Genome Atlas (TCGA) database [31]. The top 20 hub genes were extracted for interactive networks based on paired gene correlations of the stomach adenocarcinoma (STAD) cohort in TCGA (Pearson correlation coefficients). In addition, the mRNA expressions of the hub genes were also investigated between tumor and normal tissues.

Moreover, the stage-specific expression of each hub gene was also generated by GEPIA. The mRNA expressions of the hub genes of TCGA (STAD) were also retrieved from the Xena system, University of California, Santa Cruz (UCSC) for prognostic analysis [32].

### Statistical analysis

Generally, *p* value < 0.05 was defined as cut-off criterion and considered statistically significant in all cases. SPSS 17.0 (Chicago, IL, USA) and Prism 5.0 (GraphPad Software, San Diego, CA) were used for statistical analysis and illustration.

## Results

### Identification of DEGs and heat map clustering

**Fig. 1:**
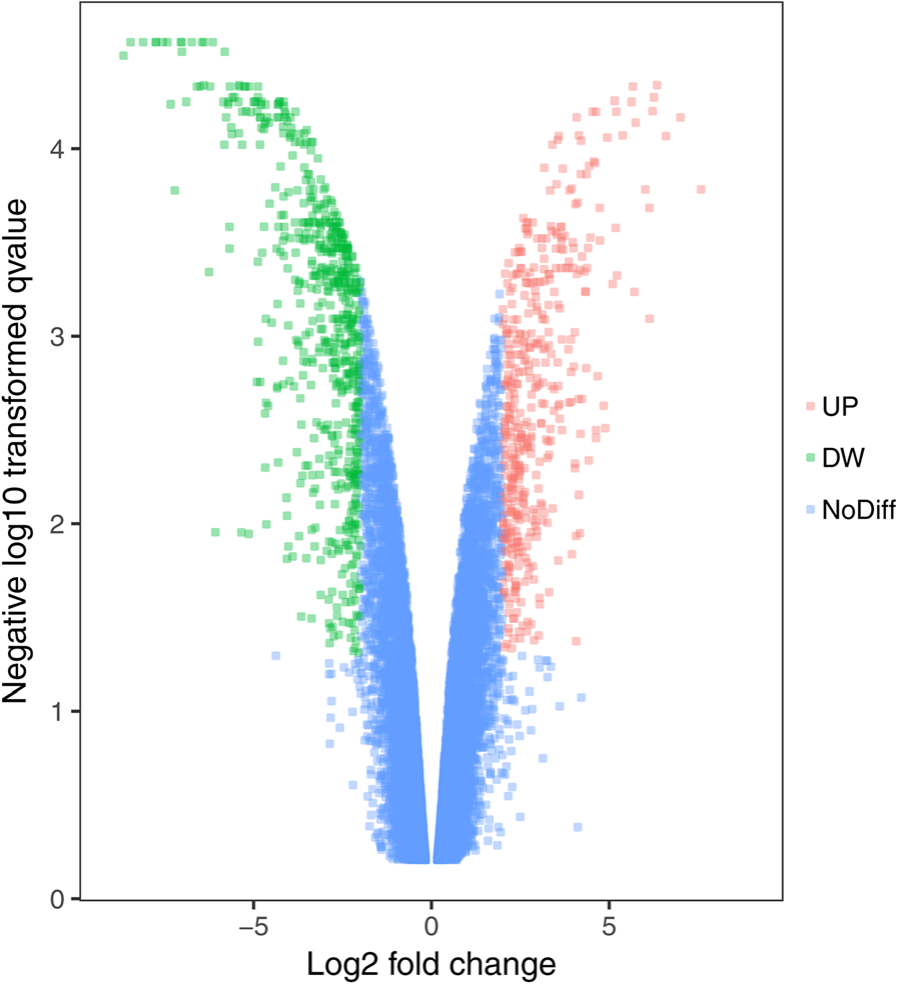
Volcano plot of the differentially expressed genes (DEGs) involved in trastuzumab-resistant gastric cancer (GC) with respect to control. The negative log10-adjusted *p* values (y-axis) were plotted against log2 fold change (log2FC) (x-axis). DEGs were identified by GEO2R. The threshold for significance was|log2FC| > 2 and adjusted *p* value < 0.05. Red, upregulated DEGs; green, downregulated DEGs

**Fig. 2:**
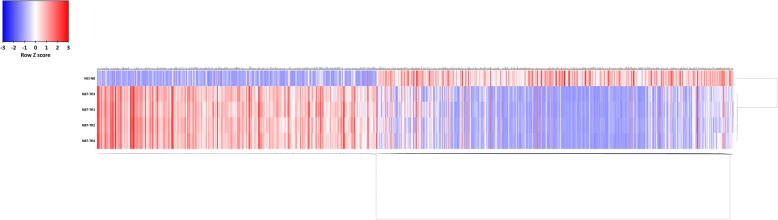
Heat map for the DEGs in trastuzumab-resistant GC cell lines. The bidirectional hierarchical clustering heat map was generated by FunRich software. The expression values were all processed by log2 fold change in prior to the heat map construction. Blue represents downregulation; red represents upregulation

A total of 849 DEGs were identified to be associated with trastuzumab resistance, with 374 genes upregulated and 475 downregulated (Fig. 1). A bidirectional hierarchical clustering heat map of the DEGs was illustrated (Fig. 2).

### GO enrichment analysis

**Fig. 3:**
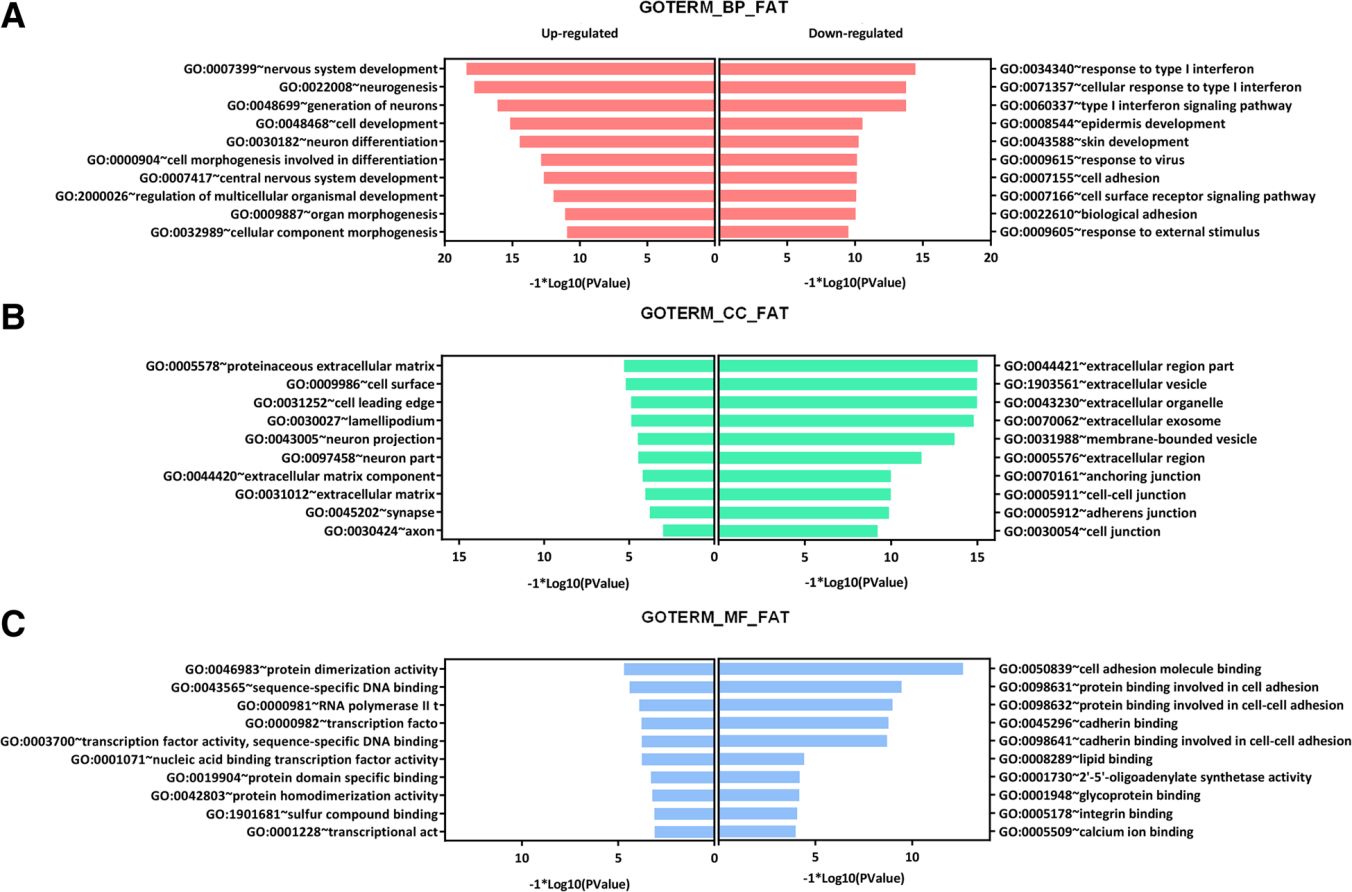
Gene ontology (GO) enrichment of the DEGs involved in trastuzumab resistance. **a** Biological function (BF) enrichment in up/downregulated DEGs. **b** Cellular component (CC) enrichment in up/downregulated DEGs. **c** Molecular function enrichment in up/downregulated DEGs

The GO enrichment analysis was conducted by the DAVID tool. A total of 193 BP terms significantly enriched, including epithelium development/cell surface receptor signaling pathway/locomotion (Table 1). A total of 23 CC terms were significantly enriched, including membrane-bounded vesicle/extracellular region part/extracellular vesicle (Table 1). A total of nine MF terms were significantly enriched, including top-ranked cell adhesion molecular binding/glycoprotein binding/growth factor binding (Table 1). Specifically, in each term, top ranked 10 most significantly enriched gene-ontologies of upregulated and downregulated DEGs were compared (Fig. 3). In BP term, nervous system development and response to type I interferon were significantly enriched in up/downregulated DEGs, respectively (Fig. 3a). In CC term, proteinaceous extracellular matrix and extracellular region part were significantly enriched in up/down regulated DEGs, respectively (Fig. 3b). In MF term, protein dimerization activity and cell adhesion molecule binding were significantly enriched in up/down regulated DEGs, respectively (Fig. 3c).

**Table 1:** Gene ontology analysis of the DEGs

Category	Term/gene function	Gene count	%	*p* value	FDR
GOTERM_BP_FAT	GO:0060429~epithelium development	112	13.25444	1.53E−17	3.01E−14
GOTERM_BP_FAT	GO:0007166~cell surface receptor signaling pathway	207	24.49704	2.44E−16	4.33E−13
GOTERM_BP_FAT	GO:0040011~locomotion	134	15.85799	4.27E−14	8.38E−11
GOTERM_BP_FAT	GO:2000026~regulation of multicellular organismal development	145	17.15976	6.52E−14	1.28E−10
GOTERM_BP_FAT	GO:0009887~organ morphogenesis	99	11.71598	9.11E−14	1.79E−10
GOTERM_CC_FAT	GO:0031988~membrane-bounded vesicle	255	30.17751	2.34E−13	3.49E−10
GOTERM_CC_FAT	GO:0044421~extracellular region part	266	31.47929	1.57E−12	2.34E−09
GOTERM_CC_FAT	GO:1903561~extracellular vesicle	206	24.3787	1.08E−11	1.61E−08
GOTERM_CC_FAT	GO:0043230~extracellular organelle	206	24.3787	1.11E−11	1.65E−08
GOTERM_CC_FAT	GO:0070062~extracellular exosome	204	24.14201	2.24E−11	3.34E−08
GOTERM_MF_FAT	GO:0050839~cell adhesion molecule binding	53	6.272189	1.39E−09	2.27E−06
GOTERM_MF_FAT	GO:0001948~glycoprotein binding	20	2.366864	1.71E−07	2.78E−04
GOTERM_MF_FAT	GO:0019838~growth factor binding	21	2.485207	1.31E−06	0.002133
GOTERM_MF_FAT	GO:0098631~protein binding involved in cell adhesion	34	4.023669	4.03E−06	0.006547
GOTERM_MF_FAT	GO:0000982~transcription factor activity, RNA polymerase II core promoter proximal region sequence-specific binding	36	4.260355	7.11E−06	0.011559

As a total of 193 biological processes (BP), 23 cellular components (CC), nine molecular functions (MF) enriched in gene ontology (GO), only the top five in each term according to the false discovery rate (FDR) value were illustrated

*DEGs* differentially expressed genes

### KEGG pathways analysis

**Fig. 4:**
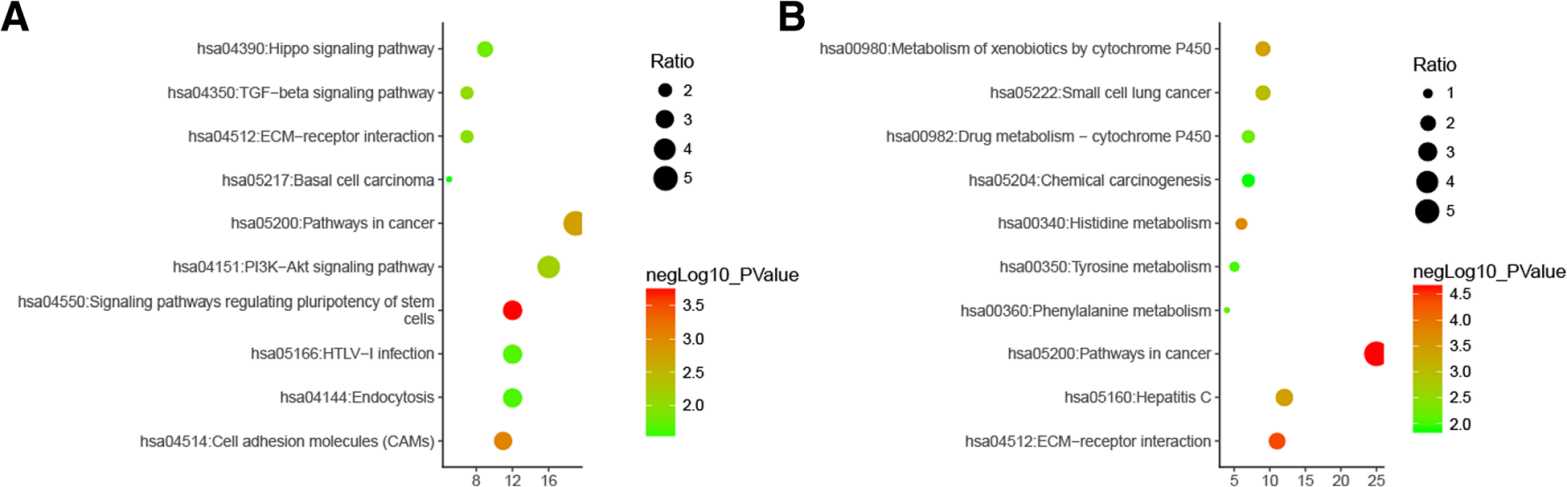
Kyoto Encyclopedia of Genes and Genomes (KEGG) analysis of the DEGs involved in trastuzumab resistance. **a** KEGG pathways in upregulated DEGs. **b** KEGG pathways in downregulated DEGs

Noteworthy, only two significant signaling pathways were identified in KEGG pathway analysis with cut-off values (*p* < 0.05, FDR < 0.05): pathways in cancer (hsa05200) and ECM-receptor interaction (hsa04512) (Table 2). The top ten enriched signaling pathways in upregulated and downregulated DEGs were illustrated, respectively (Fig. 4). Of note, no significant pathway was identified in upregulated set, and only one, the pathways in cancer (hsa5200), was identified as significant in downregulated set.

**Table 2:** KEGG pathway enrichment analysis

KEGG pathway	Gene counts	%	*p* value	FDR	Genes
hsa05200: pathways in cancer	44	5.21	5.95E−08	7.75E−05	GNG4,CCND1,STAT1,LAMB3,JUP,SMAD4,ITGA2,RUNX1,WNT5A,KIT,FGFR3,LAMA4,ITGA3,BCL2L1,FZD8,ADCY7,AXIN2,COL4A1,RAC2,COL4A6,LAMC3,SMO,LPAR5,LAMA1,RXRA,PAS1,FGF20,SLC2A1,ERBB2,ITGA6,WNT11,CDH1,TGFA,BMP2,ADCY1,FZD9,BMP4,GNG7,GNB4,KITLG,LAMC2,FGF9,F2R,LAMA5
hsa04512: ECM-receptor interaction	18	2.13	3.05E−07	3.98E−04	LAMA1,sdc1,LAMB3,ITGA6,ITGA2,ITGB4,ITGA3,LAMA4,THBS1,SV2A,COL4A1,COL6A1,COL4A6,LAMC2,SDC4,CD44,LAMC3,LAMA5

*KEGG* Kyoto Encyclopedia of Genes and Genome

*FDR* false discovery rate

### PPI network and modules

**Fig. 5:**
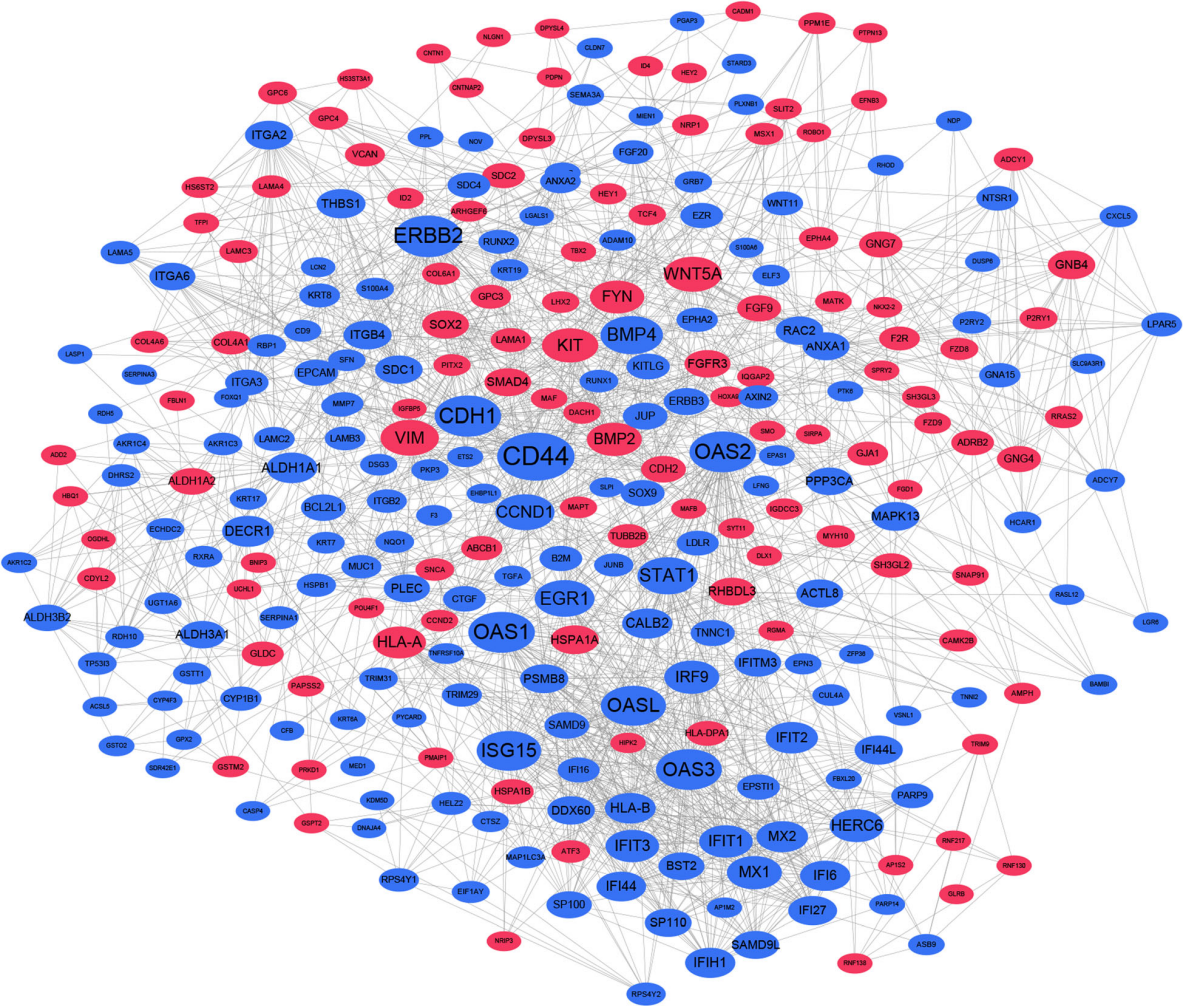
Protein-protein interaction (PPI) networks of the DEGs. Red nodes represented upregulated genes and blue nodes represented downregulated genes (nodes ≥ 5). The interaction between genes was illustrated by lines

**Fig. 6:**
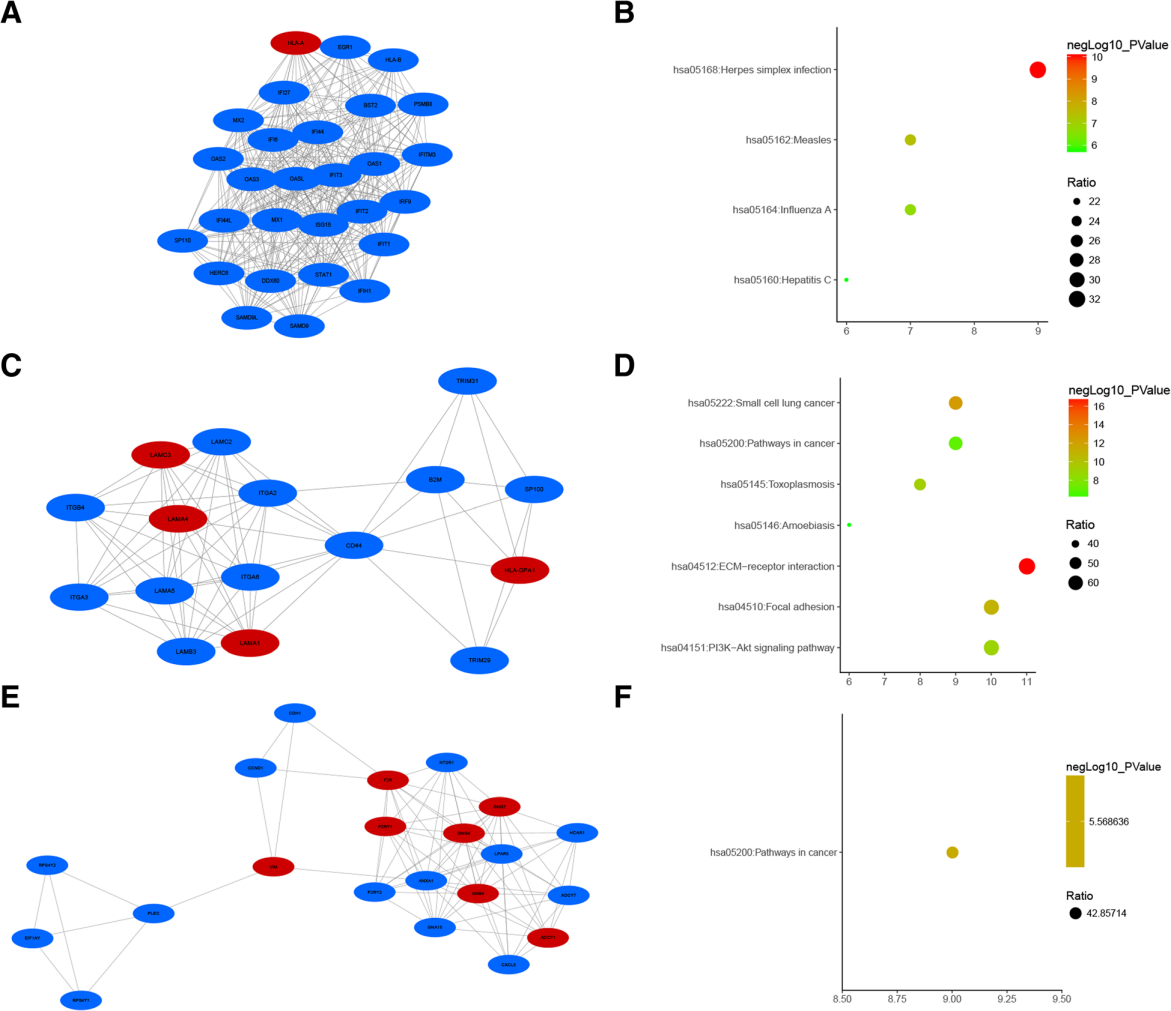
The most scored three modules with KEGG enrichment results. **a** Module-1. **b** KEGG analysis of module 1. **c** Module 2. **d** KEGG analysis of module 2. **e** Module 3. **f** KEGG analysis of module 3. Red nodes represented upregulated genes while blue nodes represented downregulated genes

Next, the PPI networks were initially obtained by the STRING database and visualized by Cytoscape with degrees of each nodes ≥ 5. A total of 291 nodes 1883 edges were included in the PPI networks (Fig. 5). The top 20 hub genes with highest degrees were determined, including CD44 molecule (CD44), erb-b2 receptor tyrosine kinase 2 (HER2), cadherin 1 (CDH1), 2′-5′-oligoadenylate synthetase 1–3 (OAS1–3), 2′-5′-oligoadenylate synthetase-like (OASL), ISG15 ubiquitin-like modifier (ISG15), bone morphogenetic protein 4 (BMP4), signal transducer and activator of transcription 1 (STAT1), early growth response 1 (EGR1), cyclin D1 (CCND1), vimentin (VIM), Wnt family member 5A (WNT5A), KIT proto-oncogene receptor tyrosine kinase (KIT), bone morphogenetic protein 2 (BMP2), interferon regulatory factor 9 (IRF9), MX dynamin-like GTPase 1 (MX1), FYN proto-oncogene, Src family tyrosine kinase (FYN), and HECT and RLD domain containing E3 ubiquitin protein ligase family member 6 (HERC6) (Fig. 5, Table 3). In addition, the top scored three modules were determined by MCODE in Cytoscape, with KEGG enrichment results (Fig. 6). Furthermore, the siRNAs of the hub genes were summarized (Additional file 1: Table S1) [33–51].

**Table 3:** Hub genes in the PPI networks

Gene symbols	Gene names	Degrees	Betweenness centrality
CD44	CD44 molecule	68	0.11543115
ERBB2	erb-b2 receptor tyrosine kinase 2	53	0.07513542
CDH1	cadherin 1	52	0.07282977
OAS1	2′-5′-oligoadenylate synthetase 1	52	0.01778379
OAS2	2′-5′-oligoadenylate synthetase 2	52	0.01854906
OAS3	2′-5′-oligoadenylate synthetase 3	51	0.01691589
OASL	2′-5′-oligoadenylate synthetase-like	50	0.01635114
ISG15	ISG15 ubiquitin-like modifier	49	0.02075018
BMP4	Bone morphogenetic protein 4	46	0.04507158
STAT1	Signal transducer and activator of transcription 1	43	0.03737542
EGR1	Early growth response 1	42	0.04589496
CCND1	Cyclin D1	41	0.03727744
VIM	Vimentin	40	0.06221522
WNT5A	Wnt family member 5A	39	0.04301014
KIT	KIT proto-oncogene receptor tyrosine kinase	37	0.03489184
BMP2	Bone morphogenetic protein 2	35	0.02672618
IRF9	Interferon regulatory factor 9	35	0.00398764
MX1	MX dynamin-like GTPase 1	35	0.00512151
FYN	FYN proto-oncogene, Src family tyrosine kinase	34	0.05255568
HERC6	HECT and RLD domain containing E3 ubiquitin-protein ligase family member 6	34	0.02783539

### Prognostic analysis and mRNA expression of hub genes

**Fig. 7:**
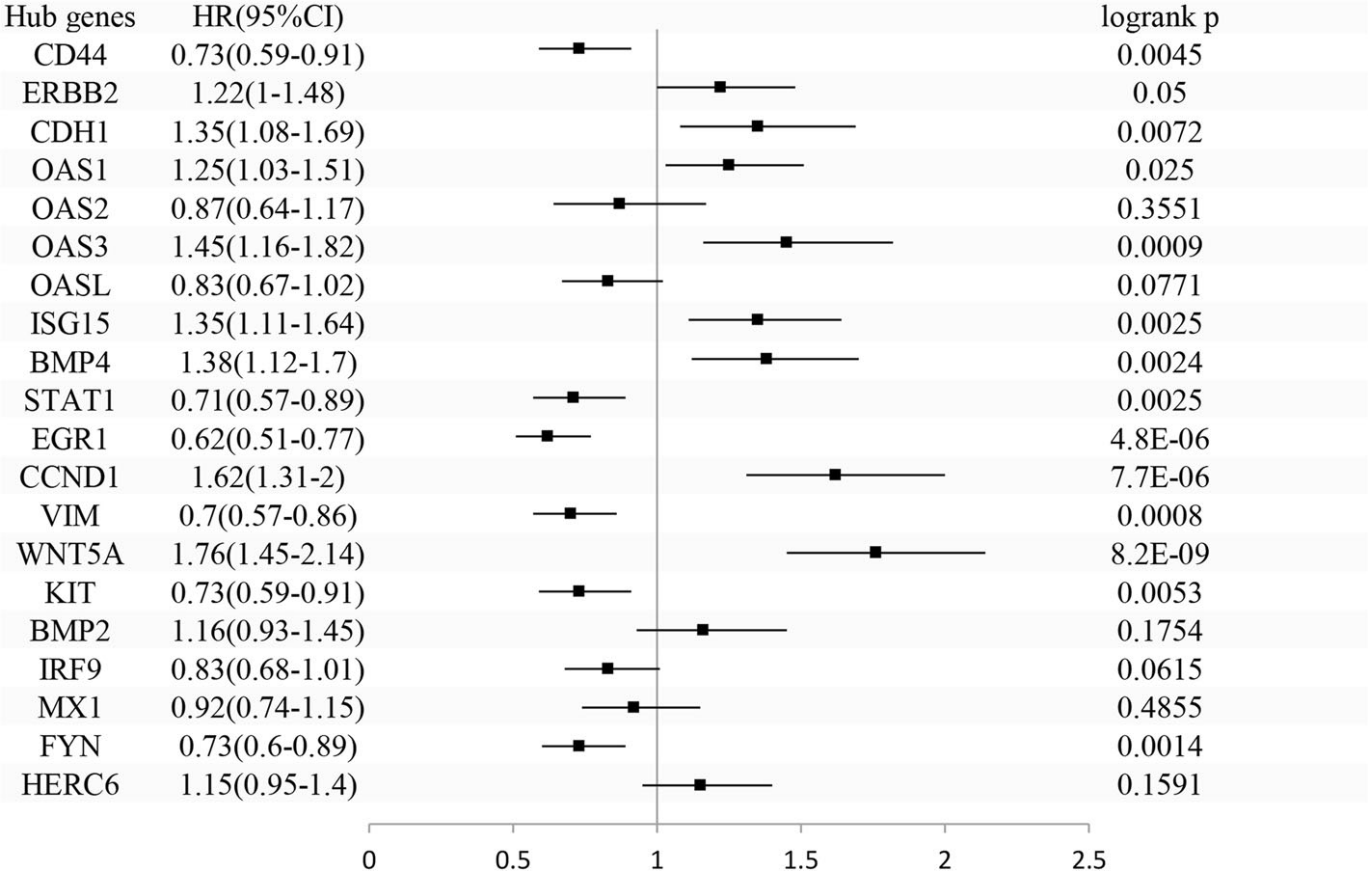
Survival plots of the prognostic values (overall survival) of hub genes involved in trastuzumab-resistant GC. The survival values of the hub genes were generated by the Kaplan-Meier (KM) plotter. The expressions of hub genes were dichotomized by optimal cutoff values. Patients number = 593. *p* values were calculated by log rank method

**Fig. 8:**
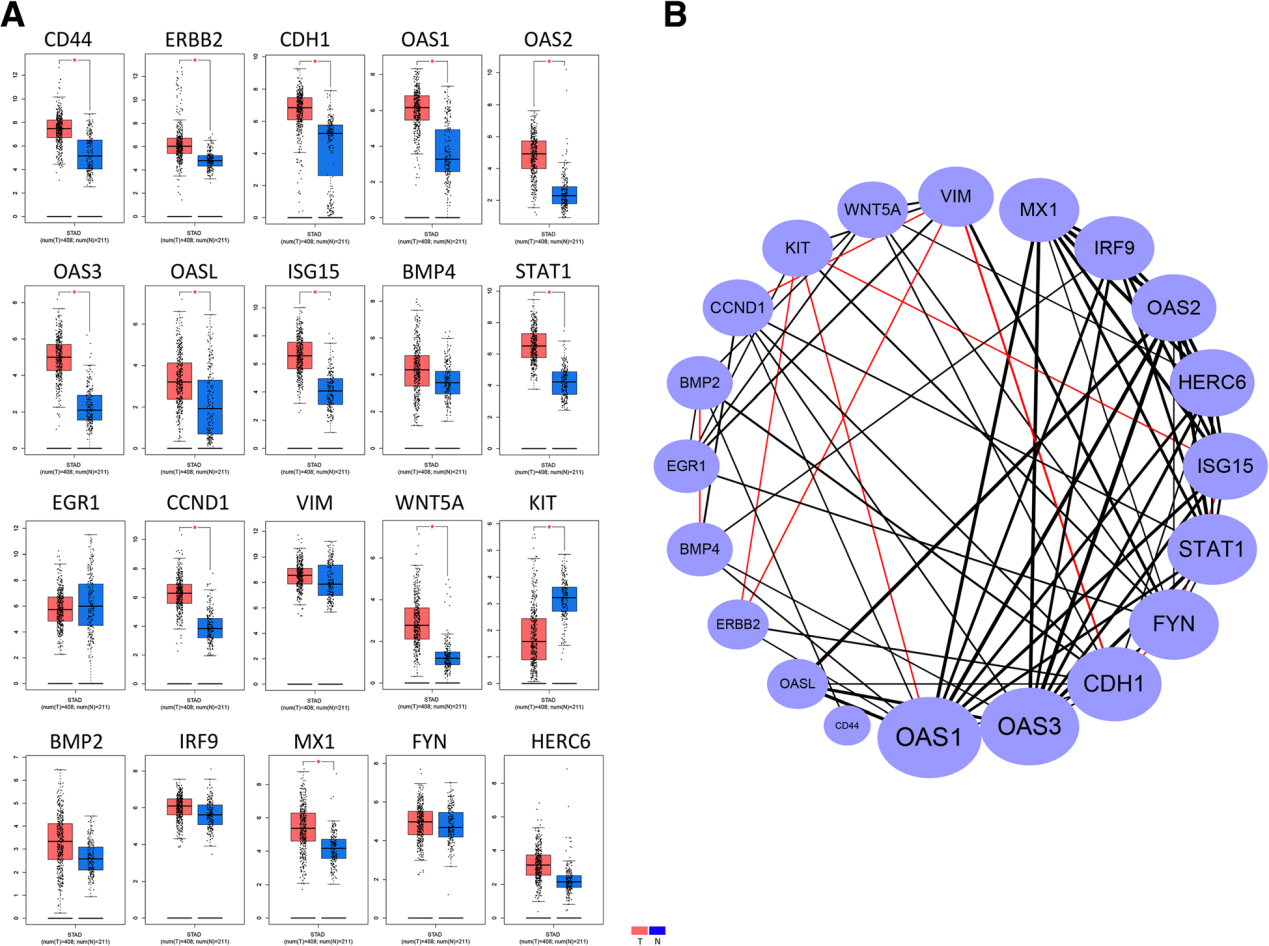
The mRNA expression and gene-gene correlation of the hub genes associated with trastuzumab-resistant GC. **a** The mRNAs expression of hub genes in tumor and normal tissues in TCGA, red: tumor, blue: normal. **b** the STAD of TCGA was calculated with Pearson’s correlation coefficient (− 1 to 1). Red line: negative correlation, black line: positive correlation. Wider line indicated higher correlation value (*p* value < 0.05)

The prognostic values of the hub genes were assessed by the KM plotter in GC. High HER2, CDH1, OAS1, OAS3, ISG15, BMP4, CCND1, and WNT5A expression levels were associated with poor OS, whereas high CD44, STAT1, EGR1, VIM, KIT, and FYN expression levels were associated with favorable OS. OAS2, OASL, BMP2, IRF9, MX1, and HERC6 were not significantly associated with OS (Fig. 7). The mRNAs expression of CD44, HER2, CDH1, OAS1, OAS2, OAS3, OASL, ISG15, STAT1, CCND1, and WNT5A were significantly upregulated in tumor while only KIT was significantly downregulated in tumor (TCGA) compared to normal (TCGA normal + GTEx normal) (Fig. 8a). Next, we further compared the mRNA expression of the hub genes between tumor (TCGA) and normal (TCGA) by the data retrieved from the Xena system. In fact, the results from the Xena (TCGA tumor vs TCGA normal) were different from GEPIA (TCGA tumor vs TCGA normal + GTEx normal). Only five hub genes (STAT1, OAS3, OAS2, CDH1, ISG15) significantly exhibited upregulation and two hub genes (KIT and EGR1) exhibited downregulation according to the thresholds (adj.*p* value < 0.05 and |logFC| > 1) (Additional file 2: Table S2). Interestingly, the gene with the most significant logFC value is KIT (logFC = − 2.11514), whereas the gene with the most significant adj.*p* value is STAT1 (adj.*p* value = 8.99E−12).

Furthermore, the mRNA expression of the hub genes (IRF9 was not available) was externally validated in GSE13861 (Additional file 3: Figure S1). Consistently, CD44, OAS3, ISG15, STAT1, and WNT5A were significantly upregulated whereas KIT was significantly downregulated in tumor compared to normal in GSE13861. Moreover, BMP4 was significantly upregulated in tumor in GSE13861. OASL, EGR1, and BMP2 were significantly downregulated in tumor in GSE13861 (Additional file 3: Figure S1). Moreover, the mRNA expression of all the hub genes in specific clinic stages had been analyzed. In fact, only CD44 (*p* = 0.0146), VIM (*p* = 1.07e−05) and KIT (0.00759) exhibited significant stage-specific expression (Additional file 4: Figure S2).

### Mechanism of hub genes correlations associated with trastuzumab resistance

To further elucidate the underlying mechanism between the DEGs, the STAD of TCGA data was employed based on GEPIA platform. Of note, 87.8% (65/74) gene-gene correlations were positive. What is more, OAS1, 3, and CDH1 featured high degrees and strong correlations with other hub genes. Additionally, VIM was negatively correlated with CCND1, HER2, and CDH1, respectively. KIT was negatively correlated with HER2, ISG15, and OAS1, respectively (Fig. 8b). Meanwhile, to investigate the potential roles of the hub genes in other target therapies, GSE19043 and GSE95414 were retrieved for external investigation (Additional file 5: Table S3). In GSE19043, none of the hub genes exhibited differential expression between gefitinib group and control, whereas in GSE95414, only six of the hub genes, including VIM, BMP2, CD44, OAS3, KIT, and WNT5A, showed slight fold change values > 1 between T-DM1-resistant cell lines and control (Additional file 6: Figure S3). In summary, the hub genes identified in this study may not be directly involved in gefitinib (EGFR inhibition, GSE95414) and T-DM1 (GSE19043) (Additional file 6: Figure S3).

## Discussion

Although the overall mortality and morbidity of GC has been declining over the decades around the globe, it is one of the most common causes for cancer-related deaths. Postoperative recurrence remains high even with curable resection and combinational chemotherapy [7–9]. Trastuzumab, the only approved treatment for GC with HER2 overexpress, had contributed to the encouraging results in GC clinical trials [13, 14]. However, secondary resistance of trastuzumab remained one of the major challenges in treatment courses. Therefore, identification of potential mechanisms and key genes underlying the acquired trastuzumab resistance could distinguish the sensitive subsets and improve overall benefits.

Generally, individual gene rarely dictate either systematic biochemical physiological actions or sophisticated multilevel network interactions. Up to now, genomic data had been stored in large matrix and processed by well-established bioinformatics pipelines for the ultimate conclusive visualization.

This study provided a systematic bioinformatics analysis of the gene expression profile, GSE77346, containing four trastuzumab-resistant cell lines and one sensitive cell line. Pathways in cancer and ECM-receptor interaction were the most significantly enriched for all DEGs. CD44, STAT1, EGR1, VIM, KIT, and FYN were associated with favorable OS while HER2, CDH1, OAS1, OAS3, ISG15, BMP4, CCND1, and WNT5A were associated with poor OS.

Mechanistically, OAS1, OAS3, and CDH1 featured highest degrees among the hub genes, diverse from the nodes (CD44, HER2, and CDH1) with highest degrees in PPI networks.

OAS1 and OAS3, which encode the key enzymes, 2′, 5′-oligoadenylate synthetase (2′5′AS), are involved in viral genome degradation and inhibits protein synthesis [52, 53]. As classic interferon target genes, OAS1 and OAS3 differ in cellular compartment, conformation, and biological functions [54]. Previously, OAS1 and OAS3 had been participated in apoptosis process [55]. Until now, only OAS3 had been associated with the HPV persistence and progression of cervical cancer [56]. No specific study unveiled the association between OAS1 and OAS3 and GC. This is the first in silico study suggesting the involvement of OAS1and OAS3 in trastuzumab-resistant GC.

CD44, a key cancer stem cell (CSC) marker, was downregulated in trastuzumab-resistant breast cancer and associated with the trastuzumab resistance in GC. [57]. Previously, high expression of CD44 correlated with downregulated HER2 in breast cancer cell lines [58]. SiRNA CD44 led to reduced internalization of trastuzumab, highlighting the involvement of endocytosis and membrane trafficking [58]. Furthermore, Bao et al. revealed that CD44 could directly bind to HER2 and increase invasiveness both in vivo and vitro [59]. Consistently, this study highlighted CD44 as the top hub gene in PPI networks of trastuzumab-resistant GC; however, the correlation between CD44 and HER2 associated with trastuzumab resistance in GC required further validation.

Noteworthy, eight of the 20 hub genes (WNT5A, BMP4, BMP2, CCND1, HER2, CDH1, KIT, STAT1) associated with trastuzumab resistance were commonly enriched in the pathways in cancer (KEGG hsa05200). Thus, the acquired resistance of trastuzumab in GC at least could be partially attributed by the progression of GC itself, if not all. Moreover, the potential impact of the mutations and fusion of the genes in the pathway in cancer on the trastuzumab resistance in GC remains largely unsolved.

In addition, for PPI networks, both degree and betweenness centrality were included for proper evaluation of hub genes. Generally, centrality is not generally equivalent to connectivity. As a local quantity, connectivity does not fully elucidate the importance of certain node in PPI networks. Thus, both connectivity and betweenness centrality were incorporated for a good measurement of hub genes in PPI networks [60].

Remarkably, ion channels, one of the major transmembrane complexes that regulate the communication between the extracellular matrix and intracellular environments, can influence the growth and invasiveness of cancer cells by altered expression or biological activities [61, 62]. In fact, ion channels could be novel molecular targets [62]. Fujimoto et al. indicated that the inhibition of ANO1, a Ca2 + -activated Cl- channel overexpressed in HER2-positive breast cancer, could lead to the transcriptional repression of HER2 in breast cancer cells with resistance to trastuzumab [63]. Another Ca2 + -permeable channel, transient receptor potential canonical 6 (TRPC6), exhibited a vital role in tumor growth, differentiation, and apoptosis with promising pharmaceutic target values [64, 65].

Recently, Huang et al. published a result focusing on the trastuzumab-resistant role of COL4A1 in GC [66]. Validation of COL4A1 in GSE77346 was one of the key steps in their study. However, GSE77346 remained far from fully explored with respect to trastuzumab resistance. In fact, new agents to be discovered against HER2 and other signaling pathways open the way to the improvement of trastuzumab therapy [67].

In breast cancer, trastuzumab remains one of the intensively studied drugs. It has been recommended as combination treatments in breast cancer [67]. In fact, mining the relationships between HER2 signaling pathway and other signaling pathways as well as the potential mechanisms provides greater insights for rational combination therapy. Currently, targets such as mTOR, PI3K, IGF-1R, Akt, HSP90, and VEGF exhibited significant clinical interests in HER2-positive breast cancer [67]. However, insightful evidences to define, refine, and optimize the use of trastuzumab in gastric cancer patients with HER2-positive remain largely lacked. Therefore, this study contributed to the understanding of trastuzumab resistance and the prognostic values of hub genes and opened the way for future research in combination therapy in gastric cancer.

Noteworthy, this was the first in silico study focusing on the bioinformatics analysis of trastuzumab resistance in GC, predicting the key genes and pathways associated with trastuzumab resistance. In addition, this study also investigated the prognostic values of key genes. However, no disease-free survival (DFS) or progression-free survival (PFS) was collected. Further clinical and experimental validation of the study findings was required.

## Conclusion

This bioinformatics analysis identified key genes and pathways as potential targets and predictors associated with trastuzumab resistance GC and further opened the way to the improvement of trastuzumab therapy in GC.

## Additional files

Additional file 1: **Table S1.** 20 hub genes with siRNA synthesizers and sequence [34–52]. (DOCX 19 kb)

Additional file 2: **Table S2.** The adj. *p* value and log fold change (logFC) of the hub genes in TCGA and GSE77346 datasets. (DOCX 19 kb)

Additional file 3: **Figure S1.** The mRNA expression of hub genes in GSE13861. (PDF 31 kb)

Additional file 4: **Figure S2.** The mRNA expression of hub genes in clinical stages in TCGA. (PDF 2878 kb)

Additional file 5: **Table S3.** Targeted drugs with corresponding GEO datasets for gastric cancer. (DOCX 18 kb)

Additional file 6: **Figure S3.** The mRNA expression and the fold change of the hub genes. (A) the mRNA expression of hub genes in GSE19043 (Gefitinib); (B) the fold change of hub genes in GSE95414. (PDF 740 kb)

## Abbreviations

BP: Biological processes
CC: Cellular components
CI: Confidential intervals
CSC: Cancer stem cell
DAVID: Database for Annotation, Visualization, and Integrated Discovery
DFS: Disease-free survival
EGFR: Epidermal growth factor receptor
FDR: False discovery rate
GC: Gastric cancer
GEO: Gene Expression Omnibus
GFP: Green fluorescent protein
GO: Gene ontology
HER2: Epidermal growth factor receptor 2
HR: Hazard ratio
KEGG: Kyoto Encyclopedia of Genes and Genomes
MCODE: Molecular Complex Detection
MF: Molecular functions
OS: Overall survival
PFS: Progression-free survival
PPI: The protein-protein interaction
RPMI: Roswell Park Memorial Institute
STAD: Stomach Adenocarcinoma
STRING: Search Tool for the Retrieval of Interacting Genes
TCGA: The Cancer Genome Atlas
