# Supplementary material for: Prediction of key genes and pathways involved in trastuzumab-resistant gastric cancer
Source: World J Surg Oncol. 2018 Aug 22;16:174. doi: 10.1186/s12957-018-1475-6 (PMC6106878; doi:10.1186/s12957-018-1475-6)
Supplement: Supplementary file 1 — Table S1. 20 hub genes with siRNA synthesizers and sequence [34–52]. (DOCX 19 kb) [file 12957_2018_1475_MOESM1_ESM.docx]

|  | siRNA synthesizer | 5’-siRNA sequences-3’ | Authors, year |
| --- | --- | --- | --- |
| CD44 | Dharmacon Inc | TTCCAGAATGGCTGATCAT | Subramaniam et al., 2007 (33) |
| HER2 | Dharmacon Inc | - | Tan et al., 2007 (34) |
| CDH1 | - | TGAGAAGTCTCCCAGTCAG | Herrero-Mendez et al., 2009 (35) |
| OAS1 | GenePharma Co.,Ltd | GCCUGGCUGAAUUACCAUTT | Zhao et al., 2016 (36) |
| OAS2 | Dharmacon Inc | AGAGGCAACUCCGAUGGUA  AAGAGAAGCCAACGUGACA  GGGAUAA GCUGAAGUUCUG  GUUGGUUUAUCCAGGAAUA | Bin et al., 2011 (37) |
| OAS3 | Dharmacon Inc.  Fisher Scientific Life Science Research | - | Lin et al., 2015 (38) |
| OASL | Shanghai Novland | AGGUGGAGCUGGUGGCAUU | Zheng et al., 2016 (39) |
| ISG15 | Dharmacon Inc | - | Chua et al., 2009 (40) |
| BMP4 | Ambion, Inc. | GGGACCAGUGAAAACUCUGtt | Xia et al., 2007 (41) |
| STAT1 | Qiagen | AACACGAGACCAATGGTGTGG | Lin et al., 2005 (42) |
| EGR1 | Invitrogen | CCAUGGACAACUACCCUAA[dT][dT] | Ogishima et al., 2005 (43) |
| CCND1 | - | GGCCUGAACCUGAGGAGCCCA | Oridate et al., 2005 (44) |
| VIM | Ambion | - | Walsh et al., 2009 (45) |
| WNT5A | Genepharma Co. | GAAGCCCAUUGGAAUAUUATT | Yang et al., 2014 (46) |
| KIT | SuperArray Bioscience Co. | - | Lefevre et al., 2004 (47) |
| BMP2 | Ambion, Inc. | GGUUUUCCGAGAACAGAUGtt | Xia et al., 2007 (41) |
| IRF9 | Invitrogen | - | Morrow et al., 2010 (48) |
| MX1 | Ambion, Inc. | CCAGCCACACGACAUUGAAUU | Toyokawa et al., 2007 (49) |
| FYN | H. Colognato | GCAGGACAGAAGATGACCT | Chen et al., 2008 (50) |
| HERC6 | B-Bridge International, Inc. | GAAAUAAGCUUUAUGCCUAUU | Arimoto et al., 2015 (51) |

Table S1. 20 hub genes with siRNA synthesizers and sequence.
