# Supplementary material for: Prediction of key genes and pathways involved in trastuzumab-resistant gastric cancer
Source: World J Surg Oncol. 2018 Aug 22;16:174. doi: 10.1186/s12957-018-1475-6 (PMC6106878; doi:10.1186/s12957-018-1475-6)
Supplement: Supplementary file 2 — Table S2. The adj. p value and log fold change (logFC) of the hub genes in TCGA and GSE77346 datasets. (DOCX 19 kb) [file 12957_2018_1475_MOESM2_ESM.docx]

|  | **adj.p-value**  **(TCGA)** | **adj.p-value**  **(GSE77346)** | **logFC**  **(TCGA)** | **logFC**  **(GSE77346)** | **P value**  **(TCGA)** | **P value**  **(GSE77346)** |
| --- | --- | --- | --- | --- | --- | --- |
| STAT1 | 8.99E-12 | 0.00026 | 1.16435 | -2.6678 | 4.50E-13 | 1E-06 |
| KIT | 2.80E-11 | 0.00568 | -2.11514 | 2.23806 | 2.80E-12 | 0.00016 |
| OAS3 | 1.66E-09 | 0.00121 | 1.19546 | -2.7664 | 2.50E-10 | 1.5E-05 |
| EGR1 | 3.11E-06 | 0.00557 | -1.21011 | -3.6258 | 6.22E-07 | 0.00015 |
| OAS2 | 5.54E-06 | 4.7E-05 | 1.11492 | -6.4973 | 1.39E-06 | 2E-08 |
| CCND1 | 3.54E-05 | 0.00854 | 0.75875 | -2.1959 | 1.06E-05 | 0.00029 |
| CDH1 | 3.86E-05 | 0.01541 | 1.22784 | -4.0645 | 1.35E-05 | 0.00072 |
| ISG15 | 5.37E-05 | 0.00491 | 1.01415 | -3.4727 | 2.15E-05 | 0.00012 |
| WNT5A | 0.00024 | 0.00306 | 0.82925 | 2.34637 | 0.00011 | 6E-05 |
| VIM | 0.0162 | 0.00048 | -0.45469 | 5.21953 | 0.0081 | 3.5E-06 |
| ERBB2 | 0.05027 | 9.5E-05 | 0.59616 | -5.3245 | 0.02765 | 2.2E-07 |
| IRF9 | 0.17105 | 0.00025 | 0.19659 | -2.6758 | 0.10263 | 8.7E-07 |
| HERC6 | 0.22614 | 0.00095 | -0.26373 | -2.5732 | 0.14699 | 1E-05 |
| OASL | 0.23104 | 0.00095 | 0.38972 | -3.4309 | 0.16173 | 1E-05 |
| OAS1 | 0.28874 | 7.1E-05 | 0.2553 | -4.0537 | 0.21655 | 1.1E-07 |
| FYN | 0.50482 | 0.00309 | -0.12361 | 2.44205 | 0.40386 | 6.1E-05 |
| BMP2 | 0.60885 | 0.00554 | -0.13824 | 2.33086 | 0.53693 | 0.00015 |
| CD44 | 0.60885 | 0.02102 | 0.12058 | -2.0203 | 0.54797 | 0.00115 |
| BMP4 | 0.68744 | 0.0001 | 0.11163 | -3.3894 | 0.65307 | 2.4E-07 |
| MX1 | 0.92983 | 5.6E-05 | 0.01986 | -6.8968 | 0.92983 | 4.2E-08 |

Table S2. The adj. p-value and log fold change (logFC) of the hub genes in TCGA and GSE77346 datasets.
