# Supplementary figures and images for: Prediction of key genes and pathways involved in trastuzumab-resistant gastric cancer
Source: World J Surg Oncol. 2018 Aug 22;16:174. doi: 10.1186/s12957-018-1475-6 (PMC6106878; doi:10.1186/s12957-018-1475-6)

# GSE13861

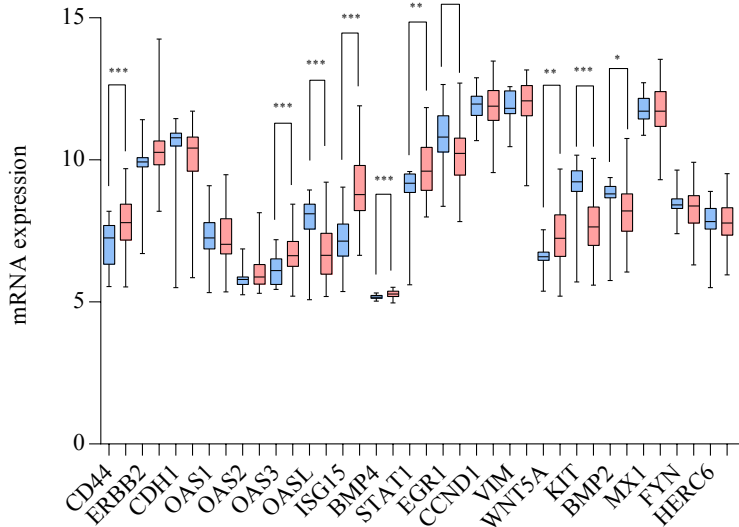

Supplement: Supplementary file 3 — Figure S1. The mRNA expression of hub genes in GSE13861. (PDF 31 kb) [file 12957_2018_1475_MOESM3_ESM.pdf]

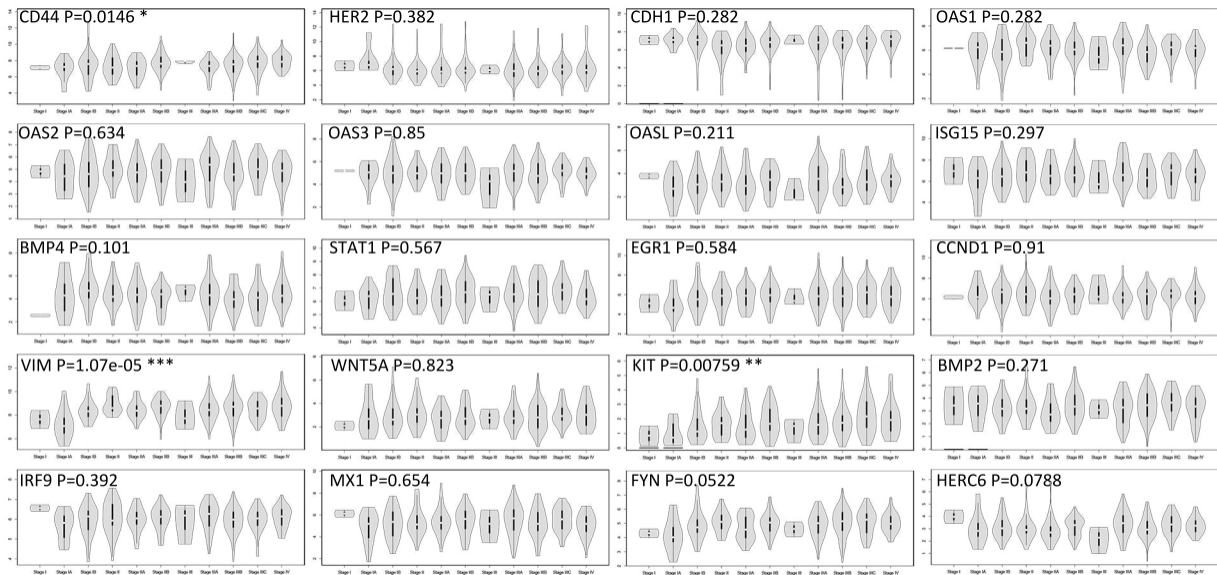

Supplement: Supplementary file 4 — Figure S2. The mRNA expression of hub genes in clinical stages in TCGA. (PDF 2878 kb) [file 12957_2018_1475_MOESM4_ESM.pdf]

A

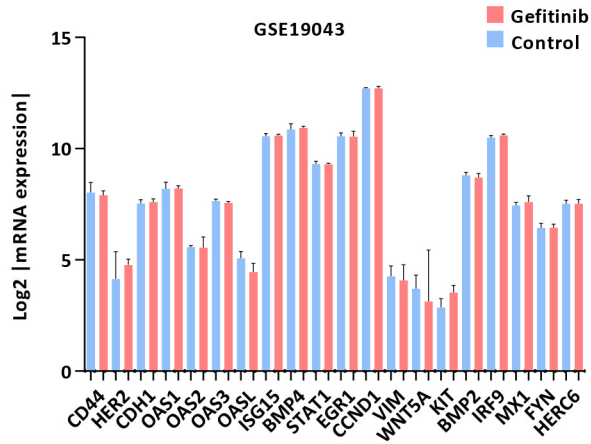

B

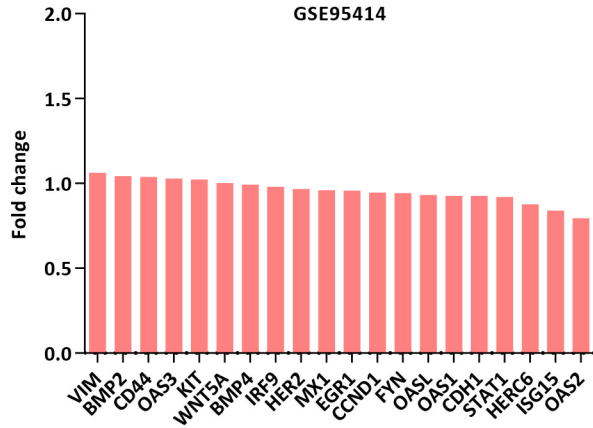

Supplement: Supplementary file 6 — Figure S3. The mRNA expression and the fold change of the hub genes. (A) the mRNA expression of hub genes in GSE19043 (Gefitinib); (B) the fold change of hub genes in GSE95414. (PDF 740 kb) [file 12957_2018_1475_MOESM6_ESM.pdf]
