# Supplementary material for: Prediction of key genes and pathways involved in trastuzumab-resistant gastric cancer
Source: World J Surg Oncol. 2018 Aug 22;16:174. doi: 10.1186/s12957-018-1475-6 (PMC6106878; doi:10.1186/s12957-018-1475-6)
Supplement: Supplementary file 5 — Table S3. Targeted drugs with corresponding GEO datasets for gastric cancer. (DOCX 18 kb) [file 12957_2018_1475_MOESM5_ESM.docx]

| **Targets** | **Drugs** | **GEO dataset availability on GC** | **Data type** | **Samples type** |
| --- | --- | --- | --- | --- |
| EGFR | Cetuximab | NA | - | - |
|  | Panitumumab | NA | - | - |
|  | Gefitinib | GSE19043 | Microarray | GTL-16 |
|  | Lapatinib | NA | - | - |
| HER2 | Trastuzumab | GSE77346 | Microarray | NCI-N87 |
|  | Lapatinib | NA |  |  |
|  | T-DM1 | GSE95414 | Microarray | NCI-N87 |
| VEGF1-3 | Regorafenib | NA | - | - |
|  | Sunitinib | NA | - | - |
|  | Sorafenib | NA | - | - |
| VEGFR-2 | Ramucirumab | NA | - | - |
| VEGF | Bevacizumab | NA | - | - |
| mTOR | Everolimus | NA | - | - |
| PARP | Paclitaxel+olaparib | NA | - | - |
| PD-L1 | Pembrolizumab | NA | - | - |

Table S3. targeted drugs with corresponding GEO datasets for gastric cancer.
